# Communication and proximity effects on outcomes attributable to sense of presence in distance bioinformatics education

**DOI:** 10.1186/1472-6920-11-10

**Published:** 2011-03-14

**Authors:** Craig Locatis, Eta S Berner, Glenn Hammack, Steve Smith, Richard Maisiak, Michael Ackerman

**Affiliations:** 1 Office of High Performance Computing and Communications, National Library of Medicine, 8600 Rockville Pike, Bethesda, Maryland 20894, USA; 2 Department of Health Services Administration, University of Alabama at Birmingham, 1675 University Boulevard, Room 534, Birmingham, Alabama 35294, USA; 3 NuPhysicia LLC, 4625 Southwest Freeway, Suite 142, Houston, Texas 77027, USA; 4 Medical Student Services, University of Alabama at Birmingham, Room 100, Volker Hall, 1670 University Boulevard, Birmingham, Alabama 25294, USA; 5 Maisiak Associates, 5444 East Grovers Avenue, Scottsdale, Arizona 85254, USA

## Abstract

**Background:**

Online learning is increasingly popular in medical education and sense of presence has been posited as a factor contributing to its success. Communication media influences on sense of presence and learning outcomes were explored in this study. Test performance and ratings of instruction and technology, factors influenced by sense of presence, are compared under four conditions involving different media and degrees of student physical presence: 1) videoconference co-located, 2) webcast co-located, 3) videoconference dispersed, and 4) webcast dispersed.

**Methods:**

Eighty one first to forth year medical students heard a lecture on telemedicine and were asked to collaboratively search a telemedicine website under conditions where the lecture was delivered by videoconference or one way streaming (webcast) and where students were either co-located or dispersed. In the videoconference conditions, co-located students could use the technology to interact with the instructor and could interact with each other face to face, while the dispersed students could use the technology to interact with both the instructor and each other. In the webcast conditions, all students could use chat to communicate with the instructor or each other, although the co-located students also could interact orally. After hearing the lecture, students collaboratively searched a telemedicine website, took a test on lecture-website content and rated the instruction and the technology they used. Test scores on lecture and website content and ratings of instruction and technology for the four conditions were compared with analysis of variance and chi-square tests.

**Results:**

There were no significant differences in overall measures, although there were on selected ratings of instruction. Students in both webcast conditions indicated they were encouraged more to follow up on their own and felt instruction was more interactive than co-located videoconferencing students. Dispersed videoconferencing students indicated the highest levels of interaction and there was evidence they interacted more.

**Conclusion:**

Results do not strongly support proximity as a sense of presence factor affecting performance and attitudes, but do suggest communication medium may affect interactivity.

## Background

Medical education is increasingly offered online, not only at a distance, but in conjunction with instruction that is face to face [1]. There is evidence that sense of presence contributes to successful online learning [2], but little is known about whether communication media affect it. Consequently, this study examined learning outcomes and ratings of instruction and technology where distance education was provided by videoconference or one way streaming (webcast) to medical students who were co-located or dispersed. The four conditions 1) videoconference co-located, 2) webcast co-located, 3) videoconference dispersed, and 4) webcast dispersed were hypothesized to offer varying degrees of presence, with sense of presence being highest when students are physically together and higher with videoconferencing since interaction is more congruent with communicating in-person. It was also hypothesized that learning outcomes and ratings of instruction and technology would be greater with higher degrees of presence, since prior research indicates sense of presence can positively affect educational outcomes and student satisfaction [2]. The study was undertaken to provide medical educators guidance in choosing among different communication and location strategies when providing instruction at a distance,

There are various definitions of presence and kinds of presence [2]. Foremost is cognitive presence, or the degree to which participants in a community of inquiry are able to construct meaning through sustained communication. Next is social presence, or the extent to which participants can project their personal characteristics and present themselves as real people. Social presence supports cognitive presence by indirectly facilitating critical thinking, but it also directly affects affective educational outcomes by making interaction enjoyable and encouraging students to remain in a course. Finally, there is teaching presence or the ways instructors develop learning activities and assessments, present content, and facilitate social and cognitive presence during a course. Presence is related to the theoretical concept of transactional distance or the degree of separation in understanding amongst teachers and students and their perceived interpersonal closeness [3-5]. Transactional distance is posited to depend on the amount of dialog and degree of flexibility in the structure of a distance course. It increases as dialog and flexibility decrease [3,4]. Transactional distance would be higher in online courses with highly structured content and only multiple choice test questions, for example, than in those allowing more freewheeling discussion through messaging and other communication modalities permitting exchange of ideas.

Researchers have argued that strategies supporting forms of presence are particularly important in computer mediated communication that primarily relies on asynchronous text messaging generally lacking the immediacy and the social and emotional cues of face to face oral communication [2,6]. They present evidence indicating interaction may be deeper and more focused in written communication but that there are higher levels of interaction that are more diverse and creative in oral communication. Although they argue the tone of the messages more than the medium affects social presence (e.g., messages that are skeptical but respectful or challenging but supportive), media are not discounted. User, content, and media characteristics have been proposed as determinants of presence [7]. Social presence, particularly, may depend on the intimacy (physical proximity, eye contact, facial expressions) and immediacy (psychological distance between communicators) a medium can convey [8].

Social presence affects the level of interaction [8] and the level of satisfaction in distance learning [2,6]. The perceived level of interaction, versus the actual level, is also associated with greater satisfaction [9]. There is evidence learners feel written communication lacks the robustness and spontaneity of face to face communication and is devoid of non-verbal cues, making it more difficult to establish trusting relationships [10]. Since computer mediated communication can lack spontaneity and richness, there is greater interest in blended learning where instruction in-person is combined with online to maximize the advantages of each [1]. There is evidence that having in-person contact with just a sub-set of students helps engender a greater sense of community among all students in a course [11] and a recent meta-analysis of studies comparing classroom and online instruction found that on average students performed better with online, but that the performance gains were largest when online was blended with classroom [12].

Videoconferencing may be acceptable when face to face or blended learning is not possible [13-15]. A meta-analysis of research on the use of video for distance education showed performance was higher when communication between students and instructors was two-way (either through student use of videoconferencing or the telephone) than when classes were broadcast one-way [16]. Studies comparing in-person and videoconferencing for clinician training [17], resident training [15,18,19], health provider re-training and clerkship training [20,21] have found performance and ratings of instruction or perceived course structure to be similar, although one study found those taught by videoconference felt there was more dialog [5]. Achievement levels also are comparable when asynchronous and synchronous video are used for pharmacy education, but there is greater satisfaction with live, interactive videoconference classes or a mix of live interactive and asynchronous ones [22].

Learning by videoconferencing is not the same as learning in-person and may add cognitive overhead to processing information [17]. Field of view is restricted by cameras, microphone placement affects one's ability to be heard, and there may be audio latencies or technology failures hampering interaction [23-25]. Camera shyness and instructor difficulty attending to students simultaneously at origination and distant sites can also make interaction difficult [15,24]. Instructors can focus more on distant learners when there is an absence of students to distract them at origination sites [26].

## Methods

Approximately equal numbers of first to forth year medical students were randomly assigned to four conditions where the mode of presentation and communication and the degree of physical proximity varied. Students received instruction by videoconference or one way streaming under conditions where they were physically co-located in a computer lab or dispersed in different rooms at their medical school. Grouping students by the full range of years in medical school helped ensure they were relatively unknown to each other.

**Figure 1:**
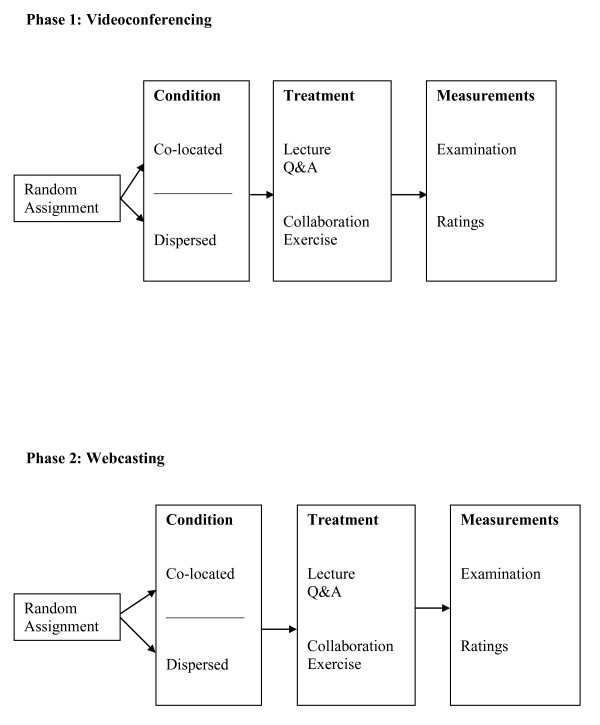
**Study Design**.

In all conditions, students listened to an instructor give a lecture on telemedicine and were then asked to collaborate doing an exercise involving searching a telemedicine website. Afterwards, they completed a seventeen item multiple choice exam on lecture and website content and scales where they rated the instruction and the communication technology. Sample test questions are shown in the Appendix and instruction and technology rating scales are depicted in Table 1. The instructional rating scale was a modified version of a longer one validated at Stanford University including items about counseling and mentoring not relevant to this study [27]. In the videoconference conditions, all students interacted with the distant instructor via videoconference, the dispersed students also could collaborate using the technology, and the co-located students could collaborate in-person. In the streaming conditions, all students interacted with the instructor via chat and could use the technology to collaborate, although the co-located students also could communicate orally. The study design is shown in Figure 1.

**Table 1:** Rating Scales

Evaluation of the Technology	Strongly Disagree	Disagree	Neutral	Agree	Strongly Agree
1. I felt I could easily communicate with other students in this session.	-2	-1	0	1	2
2. I liked using the Internet to communicate with other students during the videoconference (leave blank if you did not use the Internet for communication).	-2	-1	0	1	2
3. I prefer meeting with other students even if the instructor is not physically present.	-2	-1	0	1	2
4. I prefer communicating virtually by video conference to using email or other forms of written communication.	-2	-1	0	1	2
**Evaluation of the Presentation**					
During this presentation the presenter generally....	**Strongly Disagree**	**Disagree**	**Neutral**	**Agree**	**Strongly Agree**
1. explained the purpose of the presentation clearly and concisely.	-2	-1	0	1	2
2. explained how content applied to participants.	-2	-1	0	1	2
3. presented well organized material.	-2	-1	0	1	2
4. stayed on subject.	-2	-1	0	1	2
5. used appropriate visual aids (i.e. slides, web browser).	-2	-1	0	1	2
6. expressed respect for participants.	-2	-1	0	1	2
7. encouraged participation and interaction.	-2	-1	0	1	2
8. encouraged further learning.	-2	-1	0	1	2
9. motivated participants to follow up on their own.	-2	-1	0	1	2
10. was effective overall.	-2	-1	0	1	2

The study progressed in two stages because of funding and logistical constraints and preliminary results from the first phase were reported [28]. Data were collected on the videoconferencing conditions first and then the streaming conditions. Since the number of students who could connect simultaneously by videoconference was limited, instruction in all conditions was done in multiple sessions. The number of sessions for each condition and number of students participating in each are shown in Table 2. The number of students in each session was uneven because of difficulties in scheduling students who were volunteers receiving a twenty five dollar gift card for participating. The research plans for each stage were approved by the institutional review boards of the University of Alabama at Birmingham and the National Institutes of Health.

**Table 2:** Sessions and Students per Condition

Condition	Co-located	Dispersed
Videoconferencing	Session 1 = 10 students	Session 2 = 7 students
	Session 2 = 6 students	Session 2 = 7 students
	Session 3 = 5 students	Session 3 = 7 students
Streaming (Webcast)	Session 1 = 7 students	Session 1 = 5 students
	Session 2 = 5 students	Session 2 = 8 students
	Session 3 = 8 students	Session 3 = 6 students
Totals	Videoconferencing = 21	Videoconferencing = 21
	Streaming = 20	Streaming = 19
	Overall = 41	Overall = 40

Since the test varied to reflect changes in lecture content between the two stages, only the percentage correct for common items in each stage was used for comparison. Students were observed in the first videoconferencing stage, but problems with the telemedicine website during the webcast sessions precluded their meaningful observation. Chat records were archived for the co-located and dispersed webcast conditions in stage two, but only the interactions with the instructor for all these groups were useful. Moreover, exam questions pertaining to website content were excluded for students experiencing problems when computing exam results for comparison. A two-factor ANOVA including interaction effects was used to test for significant differences in the means between the experimental conditions. A chi-square test was used to compare differences in proportions between conditions. A follow up analysis of variance also was done comparing test performance and ratings of first and second year students to third in forth year students. Cronbach's alpha was computed to ascertain the level of internal consistency and reliability of the scales. A two-way significance level of .05 was chosen as the alpha level. SPSS Statistics (version 17.0) was the statistical software used for calculations.

## Results

The mean age of the overall sample was 23.57 (std dev = 1.71) and ranged from 20 to 28. The overall mean year in medical school was 1.99 (std dev 1.05) and ranged from 1 to 4. The dispersed students did not significantly differ from the co-located students in any of these characteristics. Students in the videoconference conditions were significantly older (mean age 24.15 vs. 23.00, p = .005), were in higher medical school years (mean 2.24 vs. 1.73, p = .03), and more likely to be male (.71 vs. .31, p = .001) than those in the streaming conditions. The racial composition of students in all conditions was similar. The instructional rating scale was highly reliable (Cronbach's alpha = .89), while the technology rating scale was moderately reliable (Cronbach's alpha = .50). The two exams were also moderately reliable (Cronbach's alpha = .42 for stage one and .41 for stage two). Examination means and standard deviations and the overall means and standard deviations of the two rating scales are shown in Table 3. The F values and significance levels resulting from and analysis of variance of these data appear in Tables 4, 5, and 6.

**Table 3:** Exam and Rating Scale Means and Standard Deviations

Condition	Exam Mean Percent Correct	SD	Instruction Mean	SD	Technology Mean	SD
Co-located Videoconference	80	.1235	1.07	.6520	.776	.5583
Co-located Webcast	85	.1260	1.38	.5188	.767	.4942
Total	83	.1249	1.23	.6038	.771	.5201
Dispersed Videoconference	83	.0787	1.14	.5142	.766	.6654
Dispersed Webcast	86	.1187	1.31	.5624	.929	.5919
Total	85	.1003	1.22	.5382	.845	.6282
All Videoconference	82	.1022	1.11	.5790	.771	.6091
All Webcast	86	.1208	1.34	.5360	.850	.5455
Grand Total	84	.1125	1.22	.5675	.810	.5762

**Table 4:** Exam Score Between Subjects Effects

Condition	Sum of Squares	df	Mean Square	F	Significance
Grouping (Co-located vs. Dispersed)	.005	1	.005	4.00	.529
Channel (Two Way Videoconferencing vs. One Way Webcast)	.028	1	.028	2.232	.139
Grouping * Channel	.000	1	.000	.030	.864

**Table 5:** Instruction Ratings Between Subjects Effects

Condition	Sum of Squares	df	Mean Square	F	Significance
Grouping (Co-located vs. Dispersed)	.001	1	.001	.004	.949
Channel (Two Way Videoconferencing vs. One Way Webcast)	1.13	1	1.13	3.55	.063
Grouping * Channel	.101	1	.101	.317	.575

**Table 6:** Technology Ratings Between Subjects Effects

Condition	Sum of Squares	df	Mean Square	F	Significance
Grouping (Co-located vs. Dispersed)	.113	1	.113	.331	.567
Channel (Two Way Videoconferencing vs. One Way Webcast)	1.18	1	1.18	.346	.558
Grouping * Channel	.146	1	.146	.429	.515

There were no significant differences in examination results or overall ratings among the different conditions. There were significant differences for three items on the instruction rating scale related to encouraging further learning, motivating students to follow up on their own, and encouraging interaction. Students in the webcast condition rated the instructor significantly higher than those in the videoconferencing condition on encouraging further learning (F = 8.25, p < .005) and motivating students to follow up on their own (F = 16.16, p < .001). Students in the co-located webcast and videoconferencing conditions also rated encouragement of participation and interaction higher than dispersed webcast students but not significantly so, while students in the dispersed videoconference condition rated this dimension of instruction significantly higher than all other groups (F = 3.88, p = .05 ANOVA interaction effect). There were no significant differences between first and second year students and third and forth year students on performance or ratings.

## Discussion

High ratings of interaction by the dispersed videoconferencing students indicates the medium's accommodation of immediate oral communication was more natural and its channeling communication so all had to participate was beneficial. Everyone was privy to the conversation, not only with the instructor but among students during the collaborative exercise. Videoconferencing sessions were observed and there was more actual interaction in the dispersed videoconferencing groups than in the co-located ones. Co-located videoconferencing students, if they interacted at all, tended to do so with the person next to them. Videoconferencing in the dispersed condition imposed participation on everyone, even if they did not speak, and may have brought everyone into closer contact than when they were physically co-located in different parts of a room.

Ironically, the webcast students' higher ratings of the instructor encouraging further learning and following up on their own might be indicators that they felt the educational experience itself was insufficient. This interpretation is cautionary, given the lack of interaction observation data for the webcast conditions, but it is supported somewhat by an examination of chat records. The fifteen total questions asked the instructor for the webcast conditions contrasts with the twenty four asked in the videoconference conditions. Although website problems precluded meaningful interaction observation, chat records show that students in the co-located webcast condition attempted to use chat to complete the collaborative website exercise (eight chat interactions for one group and three for another) even though they were physically together. The fact that the co-located webcast groups still resorted to chat indicates they were comfortable with the medium.

The lack of significant differences on other ratings and exam scores has several explanations. First, the sample size may have been too small to detect significant differences, ranging from 19 to 21 in each condition. This problem is compounded by the fact subjects were medical students and academic high performers who all did well on the examination measure. Second, the measures used were of factors sense of presence is supposed affect, not of presence itself, and the treatment involved a single session. Finally, the hypothesis made about physical co-location and two way videoconferencing offering greatest fidelity with in-person communication may be wrong, at least for certain populations, since dispersed videoconferencing students interacted more. Moreover, the students ranged in age from 20 to 28, with a mean of 23.57. Students this age may be more habituated to texting and video messaging and may not distinguish between communicating by video, chat, or face to face. Possible prior exposure to videoconferencing did not appear to be a factor given the lower and upper level medical students performed and rated instruction and technology the same.

## Conclusions

There was more interaction and higher ratings of interaction for dispersed videoconferencing students than when students were physically co-located, whether using videoconferencing or webcast. Although a definitive conclusion about actual interaction cannot be made because of lack of collaboration observation data in the webcast condition, the immediacy of interaction and the way communication is channeled to everyone by the videoconferencing technology appears more salient than physical presence [28]. This finding, combined with the lower number of questions asked the instructor during webcasts, suggests greater transactional distance and less presence for webcast and chat media. This evidence is only suggestive, given attempted use of chat by co-located groups to collaborate was interrupted by technical problems. The implications for distance learning in medical education are that, to the extent collaboration and interaction are important, technology should be used that supports oral communication and channels it amongst all members of a group. Simply bringing students physically together to collaborate is insufficient.

## Competing interests

The authors declare that they have no competing interests.

## Authors' contributions

CL designed the study, developed the instructional rating scale and took the lead in writing the manuscript.

EB assisted in study design and writing the manuscript, developed the exams and technology scale, and supervised data collection.

GH developed and delivered the course content.

SS recruited students and assisted in writing the manuscript.

RM performed the statistical analysis.

MA assisted in study design and writing the manuscript.

All authors read and approved the final manuscript.

## Appendix

### Sample Test Items

According to the glossary for health care professionals on the Telemedicine Information Exchange website, which word is defined as the following?

"The use of audio, video, and other telecommunications and electronic information processing technologies for the transmission of information and data relevant to the diagnosis and treatment of medical conditions, or to provide health services or aid health care personnel at distant sites."

A. Telematics

B. Telepresence

C. Telemedicine

D. Teleconferencing

One of the earliest telemedicine sites was:

A. LAX to UCLA Hospital

B. Logan airport to Mass General Hospital

C. USS Holland to Camp Pendleton Military Hospital

D. Carnival Cruise Ship to UTMB Hospital

## Pre-publication history

The pre-publication history for this paper can be accessed here:

http://www.biomedcentral.com/1472-6920/11/10/prepub
